# Why should we study plant sex chromosomes?

**DOI:** 10.1093/plcell/koad278

**Published:** 2024-01-02

**Authors:** Deborah Charlesworth, Alex Harkess

**Affiliations:** Institute of Ecology and Evolution, University of Edinburgh, Edinburgh EH9 3FL, UK; HudsonAlpha Institute for Biotechnology, Huntsville, AL 35806, USA

## Abstract

Understanding plant sex chromosomes involves studying interactions between developmental and physiological genetics, genome evolution, and evolutionary ecology. We focus on areas of overlap between these. Ideas about how species with separate sexes (dioecious species, in plant terminology) can evolve are even more relevant to plants than to most animal taxa because dioecy has evolved many times from ancestral functionally hermaphroditic populations, often recently. One aim of studying plant sex chromosomes is to discover how separate males and females evolved from ancestors with no such genetic sex-determining polymorphism, and the diversity in the genetic control of maleness vs femaleness. Different systems share some interesting features, and their differences help to understand why completely sex-linked regions may evolve. In some dioecious plants, the sex-determining genome regions are physically small. In others, regions without crossing over have evolved sometimes extensive regions with properties very similar to those of the familiar animal sex chromosomes. The differences also affect the evolutionary changes possible when the environment (or pollination environment, for angiosperms) changes, as dioecy is an ecologically risky strategy for sessile organisms. Dioecious plants have repeatedly reverted to cosexuality, and hermaphroditic strains of fruit crops such as papaya and grapes are desired by plant breeders. Sex-linked regions are predicted to become enriched in genes with sex differences in expression, especially when higher expression benefits one sex function but harms the other. Such trade-offs may be important for understanding other plant developmental and physiological processes and have direct applications in plant breeding.

## Teaser

Plant sex chromosomes can add to understanding of sex chromosomes generally, including regular absence of recombination near the sex-determining loci, and repeated expansion of sex-linked regions.

## Introduction

Now that genomes can be sequenced and assembly is becoming increasingly feasible, even for large genomes (albeit with challenges discussed later), genetic maps can be related to physical maps of the arrangement and nature of sequences, allowing new progress on many classical genetic problems. Among these are questions about sex chromosomes, which may evolve when cosexual species (hermaphroditic angiosperms, with “perfect” flowers, or monoecious ones, like maize) evolve separate sexed individuals. We discuss 2 questions embodied in our title: what questions make sex chromosomes interesting, and why plants are good for studying them. We focus primarily on angiosperms because their genomes tend to be smaller than those of gymnosperms whose genomes are massive and, despite the latest sequencing technologies, still highly challenging to assemble, though data are starting to be published (e.g. Gong and Filatov 2022; Liu et al. 2022). We briefly mention the advantages of studies in species with extended haploid stages, whose genomes are again often small enough for reliable sequencing and where the published studies highlight some interesting questions about sex chromosome evolution.

Plant and animal genetic sex-determining systems share interesting genomic features that demand understanding, but the angiosperms include systems that evolved more recently than the best-studied animal systems, making their evolution more accessible to study. Moreover, widely diverse flowering plant groups appear to have independently evolved separate sexes (dioecy) from cosexual ancestors, allowing consistent changes to be discerned, despite some interesting differences.

An estimated 5% to 10% of flowering plants have genetic sex determination (Yampolsky and Yampolsky 1922; Garcia et al. 2023), implying that ∼30,000 species, in taxa scattered across the angiosperms, have sex-linked genome regions. Early cytological and genetic studies (reviewed by Westergaard 1958) suggested that the sizes of completely sex-linked regions, and degrees of differentiation between the 2 members of the sex chromosome pair, must differ greatly in different angiosperm species, contrasting with the situation in the mammals, a group of similar age (Sauquet et al. 2022; Carlisle et al. 2023). More recent studies are starting to provide details of plant sex determination, and the genomic characteristics of the genome regions involved, and when they evolved, as will be described below.

**Figure 1.**
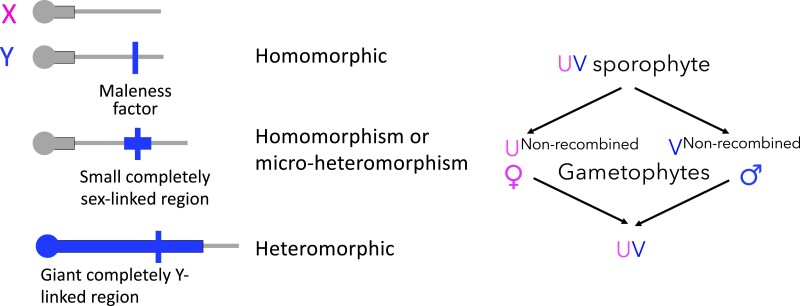
Examples of 3 different kinds of Y-linked regions observed in angiosperms with male heterogamety to illustrate concepts described in the text. The top diagram shows an X chromosome, which is similar to the ancestral state before genetic sex determination evolved; its centromere is indicated at the left (circle), with an adjoining pericentromeric region that rarely recombines. The 3 diagrams below show different types of “Y chromosomes,” with thin horizontal lines indicating recombining regions (PARs) and thick ones nonrecombining regions. The top Y is identical with the X (homomorphism), except for presence of a single male-determining factor, which is always heterozygous in males. In the second, the male-determining locus is within a small completely nonrecombining region that may include several other genes (which, again will always be heterozygous with the X in males); this situation is also likely to be classified as homomorphic because the differentiated region is a small part of the chromosome. The bottom diagram shows a strongly heteromorphic situation, with the male-determining locus in a large completely nonrecombining region across most of the chromosome arm, including the centromere, and a larger size of the arm, compared with the homologous X arm.

As shown in Table 1, some angiosperms have giant-sized sex chromosomes that resemble the ancient sex chromosomes of Eutherian mammals, birds, and Dipteran insects in being “heteromorphic,” with 1 member of the chromosome pair being repeat-rich and gene poor and sometimes heterochromatic (Fig. 1). The results described in reviews of angiosperms (Yampolsky and Yampolsky 1922; Ming et al. 2011) are probably biased toward species with heteromorphism visible to microscopists. The only heteromorphic system discovered since 1958 appears to be in *Pistacia* (Sola-Campoy et al. 2015; Palmer et al. 2022), though more will probably be discovered as genomes of further dioecious plants are sequenced or through other evidence (see below).

**Table 1.** Information about dioecious angiosperms. The table shows the range of clades in which dioecy is found, and illustrates that sex chromosome heteromorphism (species with names in bold font) is also found in distantly related taxa

Clade	Species	Heterogametic sex	Probable system	Genetic degeneration
Monocotyledon	*Phoenix dactylifera* (date palm)	Male	2 genes	Unknown
	Asparagus	Male	2 genes	Unknown
	***Dioscorea* (yam)**	Male	Unknown	Unknown
Caryophyllales	***Rumex* species (sorrel)**	Male	Unknown	Yes
	***Silene latifolia* and close relatives**	Male^a^	2 genes	Yes
	*Simmondsia chinensis* (jojoba)	Male	Unknown	Unknown
	Spinach	Male	One gene (insertion)	Unknown
Asterid	Diospyros (persimmon)	Male	One gene (duplication)	Unknown
	*Actinidia chinensis* (kiwifruit)	Male	2 genes	Unknown
	*Solanum appendiculatum*	Male	Unknown	Unknown
Rosid	***Cannabis sativa* (hemp), *Humulus lupulus* (hops)**	Male	Unknown	Yes
	***Pistacia vera***	Female	Unknown	Unknown
	***Coccinia grandis***	Male	2 genes?^b^	Unknown
	***Viscum***	Male	Unknown	Unknown
	***Hippophae rhamnoides***	Male	Unknown	Unknown
	*Carica papaya*	Male	2 genes	Unknown
	*Datisca cannabina*	Female	Unknown	Unknown
	***Vitis***	Male	2 genes	Unknown
	*Mercurialis*	Male	Unknown	Unknown
	*Populus, Salix*		One gene (duplication)	Unknown
	*Fragaria*	Female	Unknown	Unknown
	*Morus atropurpurea*	Male	Unknown	Unknown
	*Morella* (or *Myrica*) *rubra*	Female	Unknown	Unknown

Heteromorphic sex chromosome systems are probably overrepresented because, until recently, cytogenetics provided the chief evidence about plant sex chromosomes. A total of 23 species or genera are listed, of which 17 currently have genetic maps and genome sequences (further details, including references, are in Supplemental Table S1). Only 4 of these cases were not discussed in Westergaard's (1958) review; some of the species in this table are not the same as in his list, but they are in the same genus or family. Sex-determining systems with 1 or with 2 genes are found in different clades, but, for most species, the system is unknown.

^a^Female heterogamety is also known in the *Otites* clade.

^b^There is evidence for a 2-gene system in the genus *Ecballium*.

Heteromorphism and differentiation evolve when the 2 members of the sex chromosome pair do not undergo genetic recombination (at least in parts carrying the sex-determining gene or genes; regions in which the sex chromosome pair recombine, are termed “pseudo-autosomal regions” or PARs) and are not differentiated. Many species with heteromorphic sex chromosomes probably evolved separate sexes long ago. This is especially likely if high proportions of genes have been deleted, creating hemizygosity (a haploid copy number) in one sex while the other sex has diploid coverage. In the human XX female/XY male system, the Y is highly degenerated and lacks almost all genes carried on the X (Sayres and Makova 2013). In the modern bird ZZ male/ZW female system, females are similarly hemizygous for most Z-linked genes (as reviewed by Bull 1983). Among angiosperm species studied since Westergaard's (Westergaard 1958) review (Table 1), several with heteromorphic sex chromosomes show evidence for degeneration, including a *Pistacia* species with female heterogamety, whose W is partly or largely nonrecombining (Palmer et al. 2022). Genome sequencing should help to determine species' heterogametic sex and identify the degenerated sex-linked regions, if any. One major reason for studying sex chromosomes is to understand the genomic consequences of absence of recombination (which are shared with other genome regions that rarely recombine) and the processes leading to Y-X or W-Z differentiation. The degeneration just mentioned as well as the accumulation of repetitive sequences and rearrangements (see below) are consequences of a lack of recombination of the Y (and W) linked regions, whereas X (and Z) regions recombine and change from their ancestral state much less.

Plants whose haploid phase is dominant are also important for testing ideas about such genome evolutionary processes, as similar processes should operate in very different organisms. Many bryophytes have separate male and female gametophytes, and sex chromosomes were described over 100 years ago (Allen 1919; Garcia et al. 2023). They have nonrecombining regions, and, in some species, genetic PARs have been detected (McDaniel et al. 2007). As recombination occurs in the sporophyte, which is heterozygous for the sex chromosomes, both members of the sex chromosome pair are predicted to degenerate (Bull 1978), and this prediction can now be tested (as will be outlined below). To emphasize this difference from diploid XY and ZW pairs, bryophyte female- and male-determining chromosomes are termed U and V, respectively.

Many flowering plant species do not, however, have heteromorphic sex chromosomes, with detectable differentiation (the same is true in the UV systems of plants such as bryophytes with haploid-dominant life cycles, as discussed later). Their completely sex-linked regions must therefore be small (Fig. 1). Table 1 includes examples, and Westergaard's (Westergaard 1958) review included at least 13 other species or genera. Homomorphism may be more common than heteromorphism in the angiosperms (Garcia et al. 2023). Such plants may help understand why sex-determining genes are often (but not invariably) within extensive nonrecombining regions. Below, we discuss estimation of the ages of plant sex chromosomes (including for young systems that may not yet have evolved heteromorphism).

Genome sequencing now allows different types of sex-determining regions to be distinguished (reviewed by Carey et al. 2021a) and suggests when recombination stopped. One answer to our question about why plant sex chromosomes are interesting is that some homomorphic plants may be in early stages of sex chromosome evolution. This makes them excellent for identifying candidate sex-determining genes within their physically small sex-linked regions. If these genes are within nonrecombining regions, these young systems will also be ideal for discovering the mechanism(s) that created such regions (e.g. chromosomal inversions, as proposed by Lahn and Page 1999) and for describing the earliest consequences of an absence of recombination.

Among the angiosperm species in Table 1, 18 are cultivated plants. Another answer to the same question is therefore that research on sex chromosomes is important for crop breeding (Masuda and Akagi 2023). Flower development can also often be affected by environmental influences so that individuals may not always be completely male or female. So-called inconstant or leaky males express some female function under suitable conditions (Darwin 1877; Käfer and Mousset 2022), probably including genetic background influences. Females are less often inconstant, but such variability has been found in *Mercurialis annua*; in experiments in which females were grown in the absence of males, females reliably expressing male functions rapidly appeared, suggesting presence of major genetic factors (Cossard and Pannell 2021).

Nevertheless many plants exhibit clear discontinuities in gender, and major sex-determining factors can be mapped to defined genomic locations (Veltsos et al. 2019). It is often important to determine individuals' sexes in long-lived species, including many fruit crops, where only a few males are needed for pollination, or for species like asparagus, where males are desired (Harkess et al. 2015), or Ginkgo trees, where the fleshy-coated seeds smell bad, so that males are desired unless the fruits are to be used. This can be done using genotypes of variable sites in or closely linked to a sex-determining locus. Another application is to produce functionally hermaphroditic strains of fruit trees and shrubs, such as papaya and grapes, avoiding the need for separate males as pollinators. In some plants, this might involve manipulating the sex-determining system once the genes involved are known.

We first outline some recent advances in understanding the developmental properties of sex-determining genes. These are important for understanding how a situation can arise in which the 2 sexes differ at a locus with a major effect on whether individuals develop as female or male, starting from an ancestral cosexual population without such a genetic polymorphism. Then we describe how knowledge of the genes involved illuminates the observed recombination patterns in sex-linked regions.

## Sex-determining genes

Flowering plants are excellent for studying how new sex-determining genes arise. As will be seen, the mutations involved in evolving separate sexes are not just loss-of-function mutations producing females (male sterile) and males (female sterile). Both the evolutionary and developmental processes are more complex than this and often involve trade-offs between different phenotype options; male sterility may thus sometimes result from increased female functioning or vice versa.

Discovering the genes involved in plant sex-determining systems is challenging because dioecy evolved independently in different flowering plant lineages, so homology in different species is rarely a useful guide. If a species' sex chromosomes are homomorphic, there may be only a small fully sex-linked genome region, or it may have appeared too recently to have evolved sequence differentiation from its homolog, making it hard to detect or difficult to separate the sequences from the 2 sex-linked regions, as in *Amborella trichopoda* (Käfer et al. 2021). Even identifying a species' heterogametic sex is often difficult. On the other hand, heteromorphic, highly repetitive sequence densities create problems assembling highly differentiated sex chromosomes. Validating sex-determining candidates in nonmodel plants is also challenging. If these are within nonrecombining regions, genetics cannot help identify the genes, and the number of candidates for testing by transgenic experiments can be large. We discuss the technical challenges in more detail later in this review, as it is helpful to first understand important features of sex-linked regions and some of the questions relating to them.

For separate sexes to evolve from a cosexual ancestor requires at least 2 mutations, and at least 1 of them must show trade-offs. Before describing the genes that have been discovered in specific plant sex-determining systems, which seem to confirm these predictions, we first explain how the predictions emerge. Many evolutionary changes, such as changes in morphology or physiology, involve advantageous mutations spreading in populations. Adaptive characteristics such as higher activity of an enzyme, or earlier flowering, usually involve many successive changes that improve a plant function, not single mutations. Each advantageous mutation spreads throughout a population (in evolutionary terminology, becomes “fixed” in all individuals), improving the adaptation. The evolution of sex determination is an especially interesting evolutionary process, because (unlike the scenario just outlined) both sexes cannot evolve by a single mutation: to change from cosexuality to genetically controlled dioecy, at least 2 mutations are required (Charlesworth and Charlesworth 1978; Pannell and Gerchen 2018). Moreover, although mutations suppressing female or male function will rarely be advantageous, such mutations must often be involved in the evolution of dioecy, as appearance of female or male individuals involves losing functions expressed by the cosexual ancestor. This apparent puzzle is resolved if loss of female functions can allow better male functioning, or vice versa, through a developmental or other trade-off (Charlesworth and Charlesworth 1978) such that “male” plants can produce more offspring than cosexuals; Darwin called this “compensation” (Darwin 1877). Trade-offs may be common in many plant (and animal) developmental decisions, for example, in ceasing to grow more leaves and starting to flower, and are of interest for understanding plant development and functioning.

The requirement for 2 mutations to create separate females and males implies that the genes involved must suitably interact during flower development. Mutations may be advantageous only in a suitable genetic background, and this may influence the order in which mutations are able to spread in a species. In the case of sex-determining mutations, males are much more likely to spread in a population after females have first become frequent (Charlesworth and Charlesworth 1978). A final difference from many other kinds of advantageous mutations is that, if a population with genetic sex determination has evolved, at least 1 of the sex-determining mutations must have different frequencies in male and female individuals. If male heterogamety evolves, males have a male-determining factor that is absent in females (unless an X autosome balance system has evolved). Such “polymorphism” makes the genetic differences “visible” to genetic analyses. As will be explained below, either 1 or both of the 2 mutations may establish polymorphisms.

**Figure 2.**
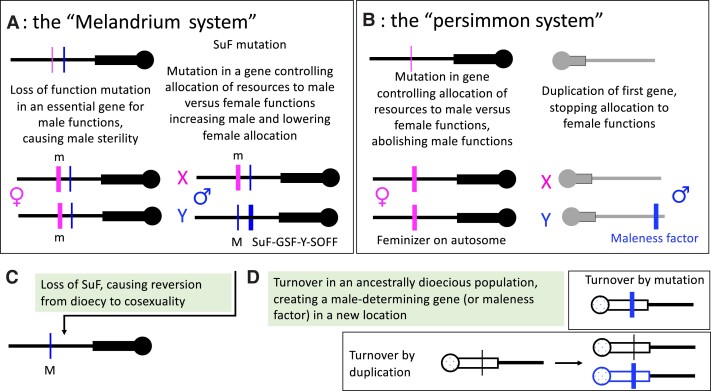
De novo evolution of separate sexes in an initially hermaphrodite or monoecious species, reversions, and turnovers. **A)** The pathway involving an initial M —> m male-sterility mutation in the hermaphroditic ancestor (in a stamen-promoting factor, or SPF), and a second mutation (or succession of mutations), denoted by SuF, GSF, or SOFF, suppresses female functions and creates males. Close linkage is predicted between the 2 mutations that define the Y-linked region, as observed. **B)** The pathway leading to the “persimmon system” in which femaleness is due to a mutation that causes active suppression of maleness (not to a loss of function mutation causing male sterility) and in which the second mutation is an unlinked duplication that creates males by suppressing the action of the allele that created females. **C)** Reversion to co-sexuality is possible under the Silene system by simple loss of the femaleness suppressor function, but in the persimmon system this is less likely (see main text), though turnover events can occur. In the pathway shown in part **D**, a new M factor can appear on any chromosome, including one that was an autosome in the ancestrally dioecious population, or in a new location on a sex chromosome. It can appear by transposition, inserting a duplicate of a progenitor gene from an ancestral location into a new one, or by in situ change of a gene, by a mutation that produces a male-determining function.

The Westergaard (1958) review of available genetic information first suggested that 2 mutations were indeed involved in sex determination in several plant species. He used “deletion mapping” of cytologically visible Y chromosome features in *Silene latifolia* to infer the location of deletions causing hermaphroditism, demonstrating the presence of a Y-linked actively femaleness suppressing factor (termed *SuF* here, though “gynoecium-suppressing factor,” or GSFY is also used). Deletions producing flowers with neither male nor female organs revealed 2 essential male function genes in other Y regions (Westergaard 1958; Kazama et al. 2016). Westergaard called this class of system the “Melandrium trigger” (as the genus *Silene* was then named Melandrium); we use “Melandrium pathway” for this pathway from co-sexuality to dioecy (Fig. 2A).

Based on maize mutations, Jones (1932) proposed a single-factor sex-determining system. This “maize trigger” did not fit observations from the dioecious plant species with segregation data available to Westergaard. However, a single-gene system was discovered in the persimmon (Akagi et al. 2014), so we term it the “persimmon system” (see Fig. 2B, and explanation below). Table 1 indicates species with 2-gene or single-gene systems, when evidence is available.

Both systems involve an actively male-promoting factor. In both, this factor defines a Y-linked genome region and triggers downstream developmental differences between male and female inflorescences and flowers and sometimes other “secondary” sex differences, such as fiber or chemical content in some dioecious species. Also, as mentioned above, females are generally expected to evolve before males in both pathways in Fig. 2. There are important differences, however. A single sex-determining factor is, by definition, a “master sex-determining factor”: its presence or absence controls whether an individual develops as a male or a female. The sex that develops in its absence is termed the “default sex,” and loss of the factor changes individuals to this sex; for example, deletion or inactivation of the maleness factor in the persimmon system can produce females. In contrast, the Melandrium system has no master sex-determining factor whose loss changes the sex and no default sex. Instead, loss of the maleness factor creates co-sexual individuals (Fig. 2C). Thus, in asparagus, mutations changing from male to female involve deletion of both Y-linked factors (Harkess et al. 2020).

### Plants with 2-gene systems and identification of the factors

Candidates for 2 primary sex-determining genes have been identified in *Actinidia* (Akagi et al. 2019), *Asparagus* (Harkess et al. 2020), and *Vitis* (Massonnet et al. 2020). Consistent with the prediction that the mutations should be in closely linked genes, these species have physically small Y-linked regions (unlike *S. latifolia*). In papaya, co-sexual domesticated strains are controlled by an hermaphrodite-determining Y, Yh, suggesting that papaya also has a Melandrium type of sex determination. A candidate Y-linked *SuF* gene (a homolog of a gene encoding the MADS-box protein *SHORT VEGETATIVE PHASE*) has been identified, whose Yh copy does not encode a complete protein (Ueno et al. 2015; Chae et al. 2021). It appears to have no X-linked homolog. No candidate has yet been identified for the mutation that created females.

In *S. latifolia*, the candidate *SuF* (or *GSFY*) is a CLAVATA *CLV3*-like gene (Kazama et al. 2022) with a highly diverged and dysfunctional X-linked homolog (termed *GSFX*), and the candidate *M* gene (whose mutation created females) is *SlWUS1*, an X-linked *WUSCHEL*-like sequence whose Y copy has been lost (Kazama et al. 2012). Disbalance of the WUSCHEL-CLAVATA feedback loop was proposed to have caused gynoecium suppression in males rather than females reflecting a loss-of-function mutation in an essential male function gene.

### The Diospyros system

The persimmon has a single-factor system (Fig. 2B; Akagi et al. 2014). Individuals carrying a factor named OGI are males, and females are the default sex. OGI is a duplication of an autosomal gene MeGI. Assuming that dioecy evolved from a co-sexual ancestor (rather than a turnover, see below), it cannot have involved this duplication alone, as individuals with only MeGI are female, and a wholly female population is impossible. More likely, the MeGI gene controlled the balance between male and female functions in the ancestral co-sexual. A mutant allele could have changed this balance to complete femaleness (rather than femaleness reflecting a loss-of function male sterility mutation). If loss of male function was favored (e.g. by allowing higher female fertility and/or if self-pollination was disfavored), females could increase in frequency; the presence of females would then favor a male-promoting mutation (Charlesworth and Charlesworth 1978). As this second mutation became more common, the advantage of the MeGI allele would also increase, and the hypothetical ancestral allele would disappear from the population, leaving OGI as the master sex-determining factor.

In principle, mutations producing females and males from an ancestral co-sexual population could occur in a single gene that controlled the balance between male and female functions. One mutation could, like MeGI, produce complete femaleness and the other complete maleness. No such systems has yet been discovered.

## Changes in sex-determining loci

### Movements between genomic locations

After separate sexes have been established, the genetic sex-determining locus may transpose to a new genome location. Related species therefore do not always have homologous sex-linked regions. In *Fragaria*, in which females are the heterogametic sex, genome regions as large as 31 kb, including multiple genes, have moved (as reviewed by Cauret et al. 2022). In the genus *Actinidia*, movements of 3 genes have occurred between different chromosomes, including the 2 sex-determining genes (Akagi et al. 2023). This strongly supports the conclusion that both the sex-determining genes that have been identified are required for the male/female difference (the Melandrium system).

Such changes can occur only if the sex-determining genes are within a physically small genome region. It may therefore be difficult to distinguish between such movements vs evolution of new single-gene systems (turnover events, described below). In *Salix* and *Populus* species, small Y-linked regions are found on different chromosomes in different species (Tuskan et al. 2012). In 2 *Populus* species, the candidate maleness factor is a partial duplication of an autosomal gene similar to the *ARR17* gene, which is important in development of female flower structures in *Arabidopsis thaliana* (Müller et al. 2020; Xue et al. 2020). The ARR17-like partial duplicates probably act as maleness factors by suppressing processes required for femaleness; indeed, experimental knockout changed the sex from male to female, the default state (Müller et al. 2020). This suggests a mechanism like the persimmon system, again with the autosomal progenitor of the duplicates being targeted by the maleness factor. Females may have arisen first, as proposed in Fig. 2B. If so, related outgroup species that have not evolved dioecy should have the ancestral state of this gene. Comparisons of the persimmon MeGI gene (or the Populus functional *ARR17* progenitor gene) with orthologs in other species may indicate how females evolved. However, most *Salicaceae* and most *Diospyros* species are dioecious, so co-sexual outgroups for such comparisons may be unavailable.

### Breakdown of dioecy, turnover events

Plant sex-determining systems are often ephemeral. Loss of separate sexes is frequently advantageous in sessile organisms, especially in colonizing situations where self-fertilization assures reproductive success when conspecific individuals or suitable pollinators are scarce (though inbreeding depression may lower progeny survival or fertility). “Secondary” co-sexuality is common in both angiosperms and bryophytes, but empirical studies of breakdown of dioecy unfortunately remain scarce. Even in the largely dioecious Salicaceae, a few species are co-sexual (Rohwer and Kubitzki 1984; Charlesworth 1985; Mirski 2014).

Under the Melandrium system, co-sexuality can readily evolve by loss of the femaleness suppressing function, *SuF*, or its deletion (see Fig. 2C above). As already mentioned, this has been achieved in papaya breeding (Storey 1953; Weingartner and Moore 2012; VanBuren et al. 2015). Co-sexuals may replace both unisexual forms. If, however, the Y chromosome is degenerated and homozygotes are inviable, as in papaya, co-sexuals may merely replace males, creating a “gynodioecious” population of X/Yh co-sexuals and X/X females (Crossman and Charlesworth 2014). Two-gene systems can also break down to androdioecy, with XY males, and XX (co-sexual) females that can produce some pollen (Charlesworth 1984), as in the genus *Mercurialis* (Gerchen et al. 2022). Turnovers in the genus *Silene* may have involved reversions to co-sexuality, and re-evolution of dioecy, including changing between XY and ZW systems (Balounova et al. 2019; Martin et al. 2019).

In contrast, a persimmon system male-determiner may be replaced with a new single-gene system in a turnover event but seems less likely to revert to the co-sexual state, because the gene that initially changed to produce females must actively suppress male functions. To revert to co-sexuality, this action must therefore be prevented. For example, a different gene involved in controlling the balance between male and female functions might produce a mutation that restores female functions. In the polyploid monoecious persimmon, *Diospyros kaki*, restoration of female functions indeed involves a different gene, a small-Myb RADIALIS-like gene, *DkRAD* (Masuda et al. 2022), and *MeGI* has evolved a epigenetic *cis*-regulatory switch that controls its expression during development, allowing appropriate production of both male and female flowers (Akagi et al. 2018; Masuda et al. 2022); *OGI* is largely silenced by a SINE-like sequence insertion into the promoter region (Akagi et al. 2018), perhaps as a secondary effect after this new system evolved.

Populations that have lost dioecy can therefore help suggest whether a plant group has a 2- or single-gene system, even if the genes have not been identified. In Table 1, 2-gene systems are suggested by such information, including for some Cucurbitaceae. Similarly, observations a gynodioecious marginal population of an otherwise dioecious *Bursera* species suggest that this species has a 2-gene system and a degenerated Y chromosome (Crossman and Charlesworth 2014).

For species with single-gene sex determination, it may be difficult to determine whether this evolved de novo from a co-sexual ancestor or by a turnover event. In principle, the persimmon or Salicaceae XY systems could have evolved when a new male-determining factor arose, replacing a preexisting system. For some taxa, it may be possible to exclude this possibility using information about related outgroup species. However, frequent changes (as in the Salicaceae) may make this problematic; the male-determining factor mentioned above may have moved its location, or dioecy could have been lost and later reevolved through independent duplications creating new, but similar, maleness factors (turnover events). This is plausible because only genes with suitable functions in development are likely to be able to evolve into maleness determiners. In fish, master sex-determining genes include various genes involved in gonad development and its hormonal control, and some genes have male-determining functions in distantly related lineages (Pan et al. 2021). Overall, with so many ways in which they can arise, the existence of single-gene systems should be unsurprising, and it will be interesting in the future to discover which of the different possible mechanisms is involved in specific taxa.

Heterogamety can also change, for example, in both poplars (Tuskan et al. 2012) and willows (Yang et al. 2021). Intriguingly, duplicates of response regulator genes are found within both Y- and W-linked regions in different Salicaceae species (Yang et al. 2021), but it is not known whether they function as sex-determining genes in both XY and ZW systems and, if so, how this is possible. Now that the direction of changes can be inferred from phylogenies and sex ratios can be assessed using sex-linked molecular markers (allowing tests for involvement of segregation distorters, which may often be involved), it will be interesting to compare these systems with theoretical models for such changes (Bull and Charnov 1977). For example, if a femaleness determining mutant can override the maleness factors in the ancestral XY + AA genotype, XY females will mate with males, creating YY individuals; this may prevent the change if the Y is degenerated and YY genotypes are inviable.

## Genetic degeneration and chromosome heteromorphism

Once formed, sex-linked regions evolve differently from autosomal regions. Young flowering plant sex-linked regions, including regions like the parts of the *S. latifolia* Y chromosome that became Y-linked only recently (Campos et al. 2016), are ideal for studying the steps involved in sex chromosome evolution. These include accumulation of repetitive transposable element (TE) and satellite sequences, which are predicted to be the first consequences of loss of recombination, because repetitive sequences in such regions do not recombine with similar sequences in other locations and cause problems in meiosis (Charlesworth et al. 1994). Enlarged Y-linked regions can therefore evolve. If high TE densities evolve, suppression of TE activity may also lead to heterochromatin formation, further suppressing recombination (Underwood et al. 2018). Although these processes are most pronounced for Y-linked regions, X-linked regions also recombine less often than autosomes because they recombine only in females. They may therefore evolve similarly, to a smaller extent, especially as TE accumulation may be sex specific, due to differences during male vs female meiosis (reviewed in Jesionek et al. 2021).

In completely sex-linked regions, the ability of natural selection to eliminate deleterious mutations is also weakened, especially for Y-linked mutations that remain heterozygous because they cannot recombine onto the X. Selection against such mutations will generally be weaker than in homozygotes, so they can rise to intermediate frequencies in the population of Y chromosomes, by genetic drift, especially as this population includes only 1 Y chromosome for every 3 X chromosomes (if the sex ratio is 1:1). Genes in Y-linked regions may therefore become nonfunctional pseudogenes or be deleted (reviewed by Bachtrog 2008). Although accumulation of repetitive sequences is not the same as degeneration, it may contribute to altered gene expression. TE mobilization (involving chromosome breaks) can also cause Y chromosome rearrangements, including deletions.

In diploid plants, regions with major gene losses are detectable by coverage analysis of male and female genome sequences: some or all X-linked genes are present in males at one-half the dosage in females (hemizygosity). Table 1 indicates species with evidence for degeneration, all of which are heteromorphic. Degeneration appears to be nearly complete in *Rumex rothschildianus* (Crowson et al. 2017) and also seems likely in *Rumex* species that have evolved X-autosome balance sex-determination systems (Smith 1969). Evidently, therefore, selection in the haploid life cycle stage cannot prevent degeneration. However, the available results do not yet reliably quantify gene losses from Y-linked regions. Most angiosperm species, even those with known heteromorphic sex chromosomes, await study using complete genome sequencing to better quantify deleterious mutations in Y-linked genes and compare with results for X-linked genes.

The nonrecombining parts of the U and V chromosomes of plants with extended haploid stages (or “haploid plants”) are expected to evolve differently from autosomal regions. Repetitive sequences are expected to accumulate but should do so similarly for U and V chromosomes because unlike Y or W chromosomes, they both recombine only in the sporophyte stage (Bull 1978). This prediction is supported by the *Ceratodon purpureus* genome sequence; the U and V are both greatly, and similarly, enriched in repetitive sequences (Carey et al. 2021b) even though U-V sequence divergence is small. Similarly, Bull also predicted that U and V chromosomes should both degenerate to similar extents.

In haploid plants, selection opposing genetic degeneration is predicted to be stronger than in diploids because the sex chromosome pair seems likely to carry many genes essential for gametophytes of either sex (Bull 1978). Detecting degeneration in UV systems is, however, difficult. In XY systems, genes present on the X and absent from the Y can be inferred to have been lost from the Y, but similar U and V degeneration prevents any such inference. In *Marchantia polymorpha*, the U and V chromosomes both carry few genes, suggesting that they are both roughly equally degenerated, as predicted. Intriguingly, however, the observations do not support the predicted slight degeneration. Between 1,900 and 3,000 genes are identified on each of the 8 *M. polymorpha* autosomes compared with only 47 apparently functional U-linked genes, 93 on the V chromosome, and 22 shared by both members of the pair, including the female-determining factor, or feminizer, which appears to be retained because it has another essential function in males during the haploid stage (Montgomery et al. 2020; Iwasaki et al. 2021). Because chromosome sizes and genes contents greatly differ, these small numbers of genes are difficult to interpret, but they suggest gene losses. This need not, however, reflect genetic degeneration but could instead indicate selection favoring non–sex-chromosomal locations for genes with essential functions in the gametophyte stages, leading to genes moving to autosomes over the vast evolutionary time since these sex-linked regions evolved. Movements from sex-linked regions to autosomes have recently been detected in bryophytes (Singh et al. 2023).

As mentioned above, the U and V sizes greatly differ in some species (Allen 1919). Such differences are unexpected and are not yet understood, but genome sequencing should help understand their causes. Current bryophyte sequences are only from species whose U and V have similar sizes. In the polyploid *Sphagnum angustifolium*, the UV pair may have evolved independently (perhaps from an ancestral monoicous state like that of some related species) (Healey et al. 2023). It is unknown how haploid plants can evolve to be monoicous and express both sex functions and whether this involves a stage in which gametophytes carry both U and V chromosomes, as suggested for the monoicous species *Ricciocarpos natans* (Singh et al. 2023). Interestingly, the *S. angustifolium* U and V are both small and derived from a larger ancestral chromosome (Healey et al. 2023). This suggests a sex chromosome miniaturization process convergent with that in *M. polymorpha*. In contrast, 2 mosses, *C. purpureus* (Carey et al. 2021b) and *Syntrichia caninervis* (Silva et al. 2021), have large, gene-rich U and V regions. These reflect fusions with at least 1 autosome (ancestral element D), perhaps due to their history of polyploidy followed by reduction in the total number of chromosomes (Lang et al. 2018). Other bryophytes have undergone fusions of an autosome with both the U and V, or with just one of them (Renner et al. 2017; Sousa et al. 2021), which could explain U-V size differences.

Fusions between autosomes and 1 or both of the sex chromosome pair also happen in angiosperms (reviewed in Westergaard 1958), including cannabis and hops, and in the genus *Viscum*, with as many as 5 Y chromosomes (Barlow and Wiens 1976). For example, X-autosome fusions have created visibly XY1Y2 karyotypes in *Rumex* species (Smith 1964; Hough et al. 2014; Grabowska-Joachimiak et al. 2015). If there is a completely Y-linked region, fusion of an autosome with an Y or X region other than the PAR can create sex chromosome heteromorphism in a completely different way from the processes described above. However, even if a completely Y-linked region exists, most loci on an autosome or autosome arm that fuses with it will not stop recombining unless recombination is absent in males. Studies of such “instant heteromorphic systems” can test for new fully Y-linked regions and determine whether proximity to fully Y-linked regions directly prevents recombination (e.g. by hindering pairing with the homologous chromosome near the breakpoint). Such newly nonrecombining regions (including young strata, see below) are also interesting for studying the time course of changes after genome regions becomes completely sex linked.

## How old are nonrecombining sex-determining regions?

Sex chromosome turnovers can make the ages of plant sex chromosomes unclear. Direct age estimates, based on estimating sequence divergence between genes within completely sex-linked regions and present on both the X and the Y (termed gametologs) are therefore important. However, the Y-linked region may be small and carry only a few genes, or degeneration of Y-linked genes may mean that few gene pairs are available, resulting in uncertain estimates. Nevertheless, enough gametolog pairs have been found to demonstrate that synonymous site divergence can be as high as 25% (or even more for some genes) in plants with heteromorphic XY pairs, including *S. latifolia* (Yue et al. 2023), and species of Cannabis and hops (Prentout et al. 2021).

Divergence is much higher between UV gametologs of the bryophyte *M. polymorpha* (Yamato et al. 2007), indicating a very ancient origination time without subsequent loss of the separate sexed state (Iwasaki et al. 2021; Singh et al. 2023). However, the haploid gametophytes of many bryophyte species produce gametes of both sexes (monoicy), indicating that sex-determining systems can change. New systems could therefore evolve in turnovers like those described above in angiosperms.

**Figure 3.**
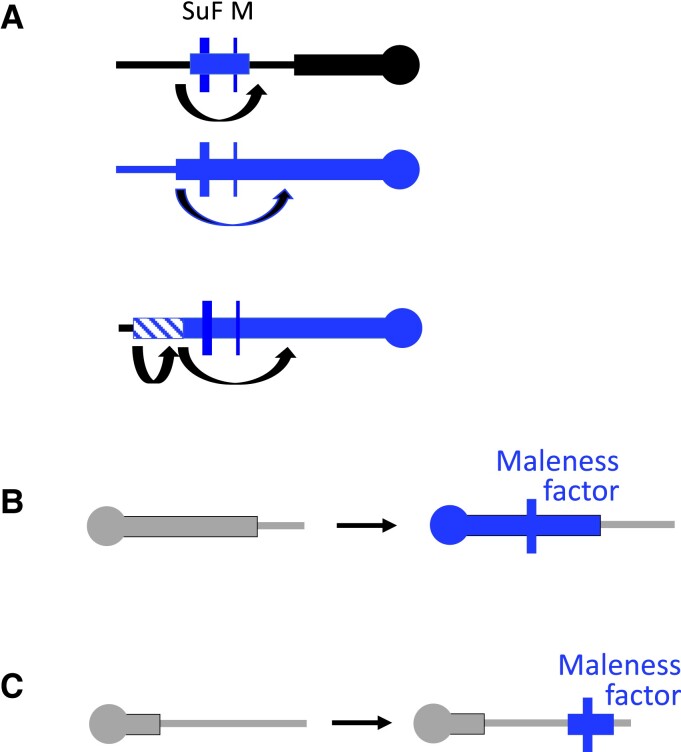
Mechanisms causing lack of recombination. As in Figs. 1 and 2, thin horizontal lines indicate chromosome arms, and thick bars that include the male-determining factors indicate complete Y linkage. **A)** Recombination suppression events creating an old evolutionary stratum. The event may involve inversion of part of the chromosome (the X or the Y, as shown), or control by a genetic recombination modifier. **B)** Evolution of a sex-determining locus in a pericentromeric region that already recombined rarely in 1 or both sexes. The processes in B and C can include turnovers causing appearance of a male-determining locus in a new genomic location. **C)** Insertion of a maleness factor creating a small Y-linked region (in a chromosome that remains morphologically similar to the X). The small Y-specific region may locally hinder recombination with the X.

We have explained that completely sex-linked regions differentiate and may become heteromorphic but not why sex-determining genes are often within physically extended nonrecombining regions. The Melandrium pathway for the evolution of separate sexes can readily explain this because crossovers between the 2 genes involved, *SuF* and *M* factors (see above), will produce neuter individuals, such as the *SuF m*/*m* genotype. This creates selection for closer linkage between the 2 genes (Fig. 3A); because *SuF* factors increase male and suppress female functions, this is termed the sexually antagonistic (SA) polymorphism hypothesis.

However, a lack of recombination does not necessarily imply that suppressed recombination has evolved. Many plant genomes include large pericentromeric regions that rarely recombine (Brazier and Glémin 2022), and sex-determining genes could evolve within them (Fig. 3B). Genetic and physical mapping has revealed that the *Rumex hastatulus* sex-linked region coincides with such a region (Rifkin et al. 2021). The same is likely for part of the *S. latifolia* Y-linked and *Pistacia* W-linked regions (Bergero et al. 2013; Palmer et al. 2022). When a large, repeat-rich Y-linked region arises in this way, the X may also be repeat rich, reflecting the ancestral state.

Duplications can create nonrecombining regions by yet another mechanism (Fig. 3C). The example of the OGI insertion in the persimmon has already been mentioned. Several *Spinacia* species have Y-linked regions in the same genome location and sharing at least 1 gene, though their sizes differ (She et al. 2022). No corresponding sequences are detected in females (Ma et al. 2022; She et al. 2023), suggesting that a duplication resulted in a new male-determining factor and Y-specific region (as diagrammed in Fig. 3D). Because the region is absent from the X, recombination cannot occur with the X. This insertion is within a large pericentromeric region like those just mentioned, instantly creating a large region that recombines rarely, at most, with the male-determining factor.

### Evolutionary strata and ongoing recombination suppression

Importantly, however, suppressed recombination must have evolved in some organisms because separate events have been discovered, creating “evolutionary strata” of Y-X divergence. Plants with such evidence for creation of new nonrecombining regions include 3 of the heteromorphic systems in Table 1, *S. latifolia* and its close relatives (Campos et al. 2016; Yue et al. 2023), whose homologous chromosome recombines in *Silene vulgaris* (Bergero et al. 2013), papaya (Wang et al. 2012), and probably the Cannabaceae (Prentout et al. 2021). It remains unclear how many dioecious angiosperms have evolved strata. The mechanism of recombination suppression is also not known. When evolutionary strata were first discovered in the human XY pair, it was suggested that chromosomal inversions could be responsible (Lahn and Page 1999). However, evidence for inversions is still scanty, and the sole example in plants is in papaya (Wang et al. 2012). A large *S. latifolia* Y-linked region is also inverted compared with the homologous chromosome of *S. vulgaris* (Bergero et al. 2013), but it is not clear when this rearrangement occurred; inversions after recombination stopped or, in the *S. vulgaris* lineage, are not excluded.

Until recently, models to explain sex-linked regions by successive changes suppressing recombination proposed that the situation was similar to the SA polymorphism hypothesis outlined above. Later mutations controlling sexually dimorphic traits might also have SA effects, so suppressed recombination might evolve if a single gene system persists long enough to allow maleness-enhancing SA polymorphisms to accumulate in genes closely or completely linked to the “primary” sex-determining locus, largely restricting the male beneficial alleles to males (Jordan and Charlesworth 2012). Polymorphisms controlling such “secondary” sexual characters create selection for closer linkage with an existing fully sex-linked region. Deletion mapping in *S. latifolia* indeed found 2 Y-linked genes whose deletion impairs (Westergaard 1948; Kazama et al. 2016), 1 of which may enhance male functions. A similar situation might explain the apparent spread of a male sterility factor after female sterility in Asparagus, the opposite of the order proposed by the Melandrium pathway (Pannell and Gerchen 2018). Studying the developmental time course of expression of Y-linked genes, and the flower bud tissues in which they are expressed, can now test whether they are likely to be involved in primary sex determination or in later reproductive stages. It will also be interesting to test whether, for genes that are expressed in both sexes, expression of Y-linked alleles differs from that of their X-linked counterparts. Such differences could reflect advantageous alleles in males with SA effects in females (or else genetic degeneration).

However, recombination might also be lost without any such SA selection or indeed any form of selection that is specifically related to sex determination. A completely sex-linked region that arises will carry deleterious mutations. Particularly if such a region is small, it may carry fewer mutations than average, favoring its initial spread throughout the Y chromosome population (Olito and Abbott 2023). Although autosomal inversions without other selective advantages are expected almost invariably to be eliminated, because of subsequent deleterious mutations within the region, some Y-linked inversions (or new Y-linked regions created by another mechanism) may escape elimination; this is because Y-linkage ensures that subsequent deleterious mutations do not become homozygous (Jay et al. 2022). If enough small inversions arise, a few lucky ones may survive and enlarge such a Y-linked region with many small strata in an apparently continuous loss of recombination process. Lenormand and Roze (2022) proposed another model, also involving successive small, lucky inversions, with each inversion favored by a special form of SA selection acting in XY heterozygotes: expression of Y-linked genes with deleterious mutations decreases, and expression of the mutation-free X-linked alleles increases in a type of dosage compensation process. This model requires asymmetry in degeneration between the sex chromosomes (see above) and therefore cannot operate in haploid plants. In a final alternative model for recombination suppression, sequence divergence in a completely sex-linked region might affect the recombination process near the boundary with the adjacent PAR, causing recombination to be continuously lost and the sex-linked region enlarged, without involving any type of selection (Jeffries et al. 2021).

These ideas may be empirically testable in dioecious flowering plants. Taxa with homomorphic sex chromosomes retained over long evolutionary times would suggest that SA polymorphisms are rarely established in plant PARs. They would also argue against the sequence divergence mechanism just outlined. Species with very recently evolved nonrecombining regions may allow tests of the other ideas. First, genome sequence assemblies can show whether new stratum formation has involved an inversion or other discontinuous change. Second, such species can be used to test whether genes in a new stratum show sex differences in expression reflecting the proposed evolution of dosage compensation alongside recombination suppression rather than after degeneration has evolved. Initial data from *S. latifolia* (Campos et al. 2016; Filatov 2022) detect no such expression pattern. On the other hand, because control of expression by sex-specific trans-acting factors is required and species in which dioecy is not very long established may not yet have such controls, most dioecious plants may be unsuitable for testing this idea; however, this argument suggests that the process is unlikely in plants.

### Dosage compensation

Degenerated Y-linked regions in diploid species may evolve dosage compensation. Degeneration causes expression in males to deviate from the optimal level (which is likely to have evolved in co-sexual ancestors of dioecious plants and which, for many genes, may still be similar in females). Also, for a few genes, expression must be balanced with that from autosomal genes. Control of expression should therefore evolve in males to restore levels nearer the optimum (reviewed in Vicoso and Charlesworth 2006). Lenormand and Roze (2022) proposed that expression of individual genes might be controlled as their Y-linked alleles acquire deleterious mutations. Plants are of special interest for studying dosage compensation because they do not have a long history of ancestors with sexually dimorphic control of gene expression (including dosage compensation). Currently, dosage compensation has been tested in few plant species (Muyle et al. 2012; Fruchard et al. 2020). Moreover, the gene sets studied include a mixture of hemizygous genes (ideal for such tests) and genes with different expression from the Y and X alleles, making the results hard to interpret. It remains unclear whether compensation has evolved in response to degeneration of sex-linked alleles because it can occur automatically for genes with mutations that decrease expression (Guo and Birchler 1994; Krasovec et al. 2019).

## Sex chromosomes and plant genomics

Genome sequences of dioecious plants across angiosperm phylogeny can help identify new sex determining genes, adding to understanding of the diverse genetic factors affecting male and female fertility across angiosperms, complementing mutant library screening within single species. The involvement of sterility mutations in some, if not all, sex-determining systems suggests that, as in fish (Pan et al. 2021), there may be many mechanisms involving different mutations that disrupt male or female functions. Identifying these possibly hundreds of independent sex-determining mutations may reveal involvement of genes not previously suspected of functioning in flower development. For instance, the sole functional annotation of the dominant SUPPRESSOR OF FEMALE FUNCTION (*SOFF*) in garden asparagus is Domain of Unknown Function 247 (Harkess et al. 2020), and orthologs of the *OGI-MEGI* suppression mechanism in persimmons had not been implicated in fertility before their sex determining actions were discovered (Akagi et al. 2014).

Contiguous assemblies of dioecious species' sex-linked regions are most useful if both members of a sex chromosome pair are included, so it is tempting to choose its heterogametic sex (XY male or ZW female) for sequencing. However, as noted above, it is often unknown whether a given dioecious species has male or female heterogamety and thus whether to sequence a male or a female individual. This is especially true for species with homomorphic sex chromosomes with small fully sex-linked regions, where cytological examination of chromosomes is not helpful unless a linkage map is available.

In species with highly degenerated Y- or W-linked regions, the heterogametic sex can be determined from a genome sequence of 2 individuals (or even just 1 of the heterogametic sex, using comparisons with autosomes). Importantly, short-read sequencing of multiple individuals of a species is no longer prohibitively expensive and can be used in analyses to identify sex-specific k-mers (Carvalho and Clark 2013) and determine heterogamety (even if no degeneration has occurred, or degeneration is slight, and coverage is similar in both sexes) by directly identifying Y- or W-linked variants. Provided that the sex-linked region is large enough and there has been enough time for variants to accumulate, this approach can yield reliable results, for example, in spinach (Ma et al. 2022; She et al. 2023). Single-nucleotide polymorphisms, the presence or absence of repetitive elements or genes, including copy number variation, can also serve to detect such associations, and analysis methods are being developed, including pangenome graphs (e.g. He et al. 2023) to represent structural differences between 2 differentiated sex chromosomes. No such analysis has yet been published for a dioecious plant, but this approach will help analyses of gene movements from autosomes into sex-linked regions and movements in the opposite direction, both of which occur in animals (reviewed in Vicoso and Charlesworth 2006) and probably also in plants (see above). Given an assembly, k-mer analysis may give a clear signal across an extensive genome region, distinguishing sex-linked from autosomal and pseudo-autosomal regions. Even assembly-free k-mer analysis can identify the heterogametic sex. There may still be an ascertainment bias toward species with at least moderate Y-X (or W-Z) differentiation across large genome regions, or “micro-heteromorphism,” but it will be much less than under complete reliance on cytogenetic observations.

It may thus be possible to assess the relative frequencies of male vs female heterogamety using living material or worldwide herbaria. Sometimes it is possible to assemble short contigs for the sex-linked regions and identify putatively sex-linked genes without expensive long-read genome assembly. Such genomic exploration of fully sex-linked regions in nonmodel species may suggest classes of genes that are enriched in sex-linked regions, test whether certain stages or tissue types or cell layers important for anther and ovule development produce sterility mutations more than others, and reveal new male sterility mutations for hybrid crop breeding.

Heteromorphic sex chromosomes in both plants and animals are often still challenging to assemble and analyze and are often excluded from nuclear genome assemblies (Pinto et al. 2023). If sex linkage is ignored in genome-wide association studies, this can cause mis-inferences, and evolutionary analyses can be compromised. For example, Y-X sequence divergence creates unusually high within-species diversity in completely sex-linked regions and closely linked sequences. Recent advances in genome assembly and scaffolding, such as >99% accurate PacBio HiFi reads, >100-kb ultra-long Oxford Nanopore reads and, more recently, >99% accurate Oxford Nanopore duplex reads (where both strands of a single DNA molecule are read) are now producing gapless genome assemblies, including the first for the human X and Y sex chromosomes (Nurk et al. 2022; Rhie et al. 2022), whereas the first human genome sequence particularly struggled to assemble either sex chromosome (International Human Genome Sequencing Consortium 2001). Such sequence assemblies will also be possible in plants.

A particular problem for sex-linked regions of nonmodel species is that (as just outlined for sex-specific k-mer analysis) the X and Y (or Z and W) versions (or haplotypes) of the region need to be distinguished. In genetical terms, the 2 haplotypes must be assembled separately, or “phased,” to study the differences between them. Fully phased assembly of sex chromosomes is difficult without knowing whether the sex-linked region in a species of interest is homomorphic (in which case, it may be small, with sequence divergence too slight for reliable phasing) or heteromorphic (in which case 1 haplotype in the region may be repetitive and/or rearranged). Manual curation, although laborious, can succeed in resolving the 2 haplotypes. Sequencing both sexes of a species can assist phasing, especially using multiple individuals of each sex. For individual gene sequences, this can provide Y-X divergence time estimates, even when sequence divergence is slight (Campos et al. 2016). However, haplotype switching and chimerism in high-throughput sequencing may hinder assembly of highly contiguous sex chromosomes. With sequences from multiple individuals within a species, it will become possible to detect differences in Y chromosomes of different individuals, including copy number variation, variation in the sizes of arrays such as satellite arrays, and large deletions in highly degenerated regions, causing variable Y or W chromosome sizes (Puterova et al. 2018).

We already mentioned the value of identifying sex-determining genes and sex-linked markers to allow early selection of individuals of the desired sex in long-lived dioecious crop plants. In transformable dioecious species, there may be opportunities for improvement of desired functions. For example, in a fruit crop species, rather than using genetic markers to remove the sex that is needed only for pollination, sex ratio distorters could be identified and used to modify sex ratios. Moreover, since 1 member of a sex-linked region is sex specific (Y-linked in species with male heterogamety or W-linked under female heterogamety), transgenes could be placed in a fully sex-linked region to ensure transmission exclusively through females, minimizing escape though the transgenic plant’s pollen.

## Conclusions

Rapid advances are currently being made in discovering sex-linked regions in dioecious plant genomes. Detailed studies of more species may reveal such regions in diverse species. This may distinguish species with extensive sex-linked regions and without them, and those whose sex-linked regions coincide with ancestrally rarely recombining pericentromeric regions, from others in which recombination has become suppressed as dioecy evolved. It may soon become possible to classify the sex-determining system of flowering plants into those with 2-gene or 1-gene systems. The species so far studied may not, of course, include all possible types of sex-determining system. For example, no system has yet been discovered in which 2 mutations within the same gene create the 2 sexes.

With defined questions in mind, future studies can choose the most suitable species for study. For instance, the Cucurbitacae are a large group of important crops, and many species have genetic sex-determination (while others are monoecious). They may have sex chromosomes of differing ages, but it is currently not even known whether dioecy evolved several times independently in this plant family. Although Cucurbitacae often have large genomes, approaches can now sequence and assemble them, as just discussed. Flowering plants are ideal for studying the time course of genome evolution and evolution of nonrecombining regions. If a succession of inversions is responsible, as recently proposed (Jay et al. 2022; Lenormand and Roze 2022), they should be readily detected in young evolutionary strata where later rearrangements have not yet obscured earlier ones. In addition, plants offer multiple independent instances of loss of recombination, at different times in the past, allowing studies of accumulation of TE and other repetitive sequences and genetic degeneration. Current data, although interesting, have served more to define the interesting questions, and new advances in sequencing hold promise that future studies will answer some of them.

## Supplemental data

The following materials are available in the online version of this article.

**Supplemental Table S1.** Information about dioecious angiosperms, showing the range of clades in which dioecy is found, and illustrating that sex chromosome heteromorphism (species with names in bold font) is also found in distantly related taxa.

## Supplementary Material

koad278_Supplementary_Data

## Dive Curated Terms

The following phenotypic, genotypic, and functional terms are of significance to the work described in this paper:

ARR17 Gramene: AT3G56380
ARR17 Araport: AT3G56380

flower AmiGo: PO:0009046

initial AmiGo: PO:0004011
