# Supplementary material for: Why should we study plant sex chromosomes?
Source: Plant Cell. 2024 Jan 2;36(5):1242–56. doi: 10.1093/plcell/koad278 (PMC11062472; doi:10.1093/plcell/koad278)
Supplement: koad278_Supplementary_Data [file koad278_supplementary_data.pdf]

Supplemental Table 1. Information about heterogamety in dioecious flowering plants, including selected modern references showing species that now have genetic and genomic resources. The taxonomic groups to which the species belong are shown, illustrating the wide distribution of dioecy in angiosperms. Where information is available, the presence of sex chromosome heteromorphosm is indicated. A summay of these data is in Table 1 of the main text.

Species	Family	Order etc.	Sex of relatives	Heterogamety	Evidence for heteromorphism	Genetic map?	Genome sequence?	Probable system (when known)	Evidence for degeneration	In Westergaard's 1958 rreview	Selected modern references (full references below)
**Heteromorphic**											
Cannabis sativa , *Humulus lupulus* (hops)	Cannabaceae	Rosales	Often dioecious	Male	Fusions with autosomes in some species	Yes	Yes	Unknown	Yes	Yes	(Divashuk et al., 2014; Faux et al., 2016; Prentout et al., 2021; Havill et al., 2023
Rumex species	Polygonaceae	Caryophyllales	Mostly dioecious	Male	Fusions with autosomes in some species	Yes (R.hastatulus)	Yes	Unknown	Yes	Yes	Hough et al., 2014; Jesionek et al., 2021
Silene latifolia and close relatives	Caryophyllaceae	Caryophyllales	Many hermaphrodite	Male †	Y > X	Yes (X chromosome)	Yes	2 genes	Yes	Yes	Fujita et al., 2011; Bergero et al., 2013; Yue et al., 2023
Pistacia species	Anacardiaceae	Sapindales, Rosids	Often dioecious	Female	Heteropycnosis, genetic map	Yes	Yes	Unknown	—	No	Sola-Campoy et al., 2015; (Palmer et al., 2022
Coccinia grandis	Cucurbitacea	Cucurbitales, Rosids	Many dioecious	Male	Y > X			2 genes	—	Yes (also Ecballium and Bryonia)	Sousa et al., 2017
Viscum	Santalaceae	Santalales	Mostly dioecious		Fusions with autosomes in some species			Unknown	—	Yes	(Barlow and Wiens, 1976
Hippophae rhamnoides	Eleagnaceae	Rosales	Small family	Male	Y < X			Unknown	—	No	Truţă et al., 2010; Puterova et al., 2017
**Probably homomorphic**											
Phoenix dactylifera (date palm)	Arecaceae	Arecales (monocotyledon)	Often dioecious	Male	—	Yes	Yes	2 genes	—	Yes (other palm species)	Torres et al., 2018
Simmondsia chinensis (jojoba)	Simmondsiaceae	Caryophyllales	(No other species)	Male	—			Unknown	—	No	Al-Dossary et al., 2021
Carica papaya	Caricacea	Brassicales	Mostly dioecious	Male	—	Yes	Yes	2 genes	At most minor	Yes	Liu et al., 2004; Wang et al., 2012
Datisca cannabina	Datiscaceae	Cucurbitales, Rosids	Dioecious (small family)	Female	—			Unknown	—	Yes	
**Vitis**	Vitaceae	Vitaceles (Rosids)	Others dioecious	Male	—	Yes	Yes	2 genes	No	Yes	Picq et al., 2014; Massonnet et al., 2020
Diospyros (persimmon)	Ebenaceae	Ericales (Asterids)	Mostly dioecious	Male	—	Yes	Yes	One gene (duplication)	—		Akagi et al., 2014; Masuda et al., 2022
Mercurialis	Euphorbiaceae	Malpighiales (Rosids)	Many monoecious	Male	—	Yes	Yes	Unknown	No	Yes	Gerchen et al., 2022
Populus, Salix	Salicaceae	Malpighiales (Rosids)	Mostly dioecious	Male, female	—	Yes	Yes	One gene (duplication)	No	Yes	Zhou et al., 2019; Almeida et al., 2020; Müller et al., 2020; Xue et al., 2020; Zhou et al., 2020; Li et al., 2021; Sanderson et al., 2021; Wang et al., 2022
Asparagus	Asparagaceae	Asparagales (monocotyledon)	Small family	Male	—	Yes	Yes	2 genes	No	Yes	(Harkess et al., 2020
Fragaria	Rosaceae	Rosales (Rosids)	Mostly dioecious	Female	—	Yes	Yes	Unknown		Yes	Tennessen et al., 2016
Spinach		Amaranthaceae, Caryophyllales	Dioecy, monoecy	Male	—	Yes	Yes	One gene (insertion)	No	Yes	She et al., 2022
Actinidia chinensis (kiwifruit)	Actinidiaceae	Ericales, Asterids	Mostly dioecious	Male	—	Yes	Yes	2 genes	No	Yes	Akagi et al., 2019
Morus atropurpurea	Moraceae	Rosales (Rosids)	Mostly dioecious	Probably male	Fst between the sexes	No	Yes	Unknown	—	No	Dai et al., 2023
Amborella trichopoda	Amborellaceae	Amborellales	Small family	Female	—	No	Yes	Unknown	—	No	Käfer et al., 2021
Solanum appendiculatum	Solanaceae	Solanales (Asterids)	Some others dioecious	Probably male	—	No	Yes	Unknown	No	No	Wu et al., 2021
Morella (or Myrica) rubra	Myricaceae	Fagales	Mostly dioecious	Probably Female	—	Yes	Yes	Unknown	—	No	Jia et al., 2019
Dioscorea (yam)	Dioscoreaaceae	Dioscoreales (monocotyledon)	Mostly dioecious	Male	—	Yes	Yes	Unknown	—	Yes	Tamiru et al., 2017; Cormier et al., 2019; Ngwe and Siljak-Yakovlev, 2023

† Female in Otites group, with unclear hetermorphism status

Full references

Akagi, T., Henry, I.M., Tao, R., and Comai, L. (2014). A Y-chromosome–encoded small RNA acts as a sex determinant in persimmons. Science **346,** 646-650.

Akagi, T., Pilkington, S.M., Varkonyi-Gasic, E., Henry, I.M., Sugano, S.S., Sonoda, M., Firl, A., McNeilage, M.A., Douglas, M.J., Wang, T., Rebstock, R., Voogd, C., Datson, P., Allan, A.C., Bepp, K., Kataoka, I., and Tao, R. (2019). Two Y-chromosome-encoded genes determine sex in kiwifruit. Nature Plants **5,** 801–809.

Al-Dossary, O., Alsubaie, B., Kharabian-Masouleh, A., Al-Mssallem, I., Furtado, A., and Henry, R.J. (2021). The jojoba genome reveals wide divergence of the sex chromosomes in a dioecious plant. The Plant Journal **108,** 1283-1294.

Almeida, P., Proux-Wera, E., Churcher, A., Soler, L., Dainat, J., Pucholt, P., Nordlund, J., Martin, T., Rönnberg-Wästljung, A.-C., Nystedt, B., Berlin, S., and Mank, J.E. (2020). Genome assembly of the basket willow, *Salix viminalis* , reveals earliest stages of sex chromosome expansion. BMC Biology **18**.

Barlow, B.A., and Wiens, D. (1976). Translocation heterozygosity and sex ratio in *Viscum fischeri* . Heredity **37,** 27-40.

Bergero, R., Qiu, S., Forrest, A., Borthwick, H., and Charlesworth, D. (2013). Expansion of the pseudoautosomal region and ongoing recombination suppression in the *Silene latifolia* sex chromosomes. Genetics **194,** 673-686.

Cormier, F., Lawac, F., Maledon, E., Gravillon, M.C., Nudol, E., Mournet, P., Vignes, H., Chair, H., and Arnau, G. (2019). A reference high-density genetic map of greater yam (Dioscorea alata L.). Theor. Appl. Genet. **132,** 1733-1744.

Dai, F.W., Zhuo, X.K., Luo, G.Q., Wang, Z.J., Xu, Y.J., Wang, D., Zhong, J.W., Lin, S., Chen, L., Li, Z.Y., Wang, Y., Zhang, D.Y., Li, Y.Y., Zheng, Q.Y., Zheng, T.C., Liu, Z.J., Wang, L., Zhang, Z.Y., and Tang, C.M. (2023). Genomic resequencing unravels the genetic basis of domestication, expansion, and trait improvement in *Morus atropurpurea* . Advanced Science.

Divashuk, M., Alexandrov, O., Razumova, O., Kirov, I., and Karlov, G. (2014). Molecular cytogenetic characterization of the dioecious *Cannabis sativa* with an XY chromosome sex determination system. PLOS ONE **9,** e85118.

Faux, A.M., Draye, X., Flamand, M.C., Occre, A., and Bertin, P. (2016). Identification of QTLs for sex expression in dioecious and monoecious hemp (Cannabis sativa L.). Euphytica **209,** 357-376.

Fujita, N., Torii, C., Ishii, K., Aonuma, W., Shimizu, Y., Kazama, Y., Abe, T., and Kawano, S. (2011). Narrowing down the mapping of plant sex-determination regions using new Y chromosome-specific markers and heavy-ion beam irradiation-induced Y deletion mutants in *Silene latifolia* . G3 **2,** 271-278.

Gerchen, J., Veltsos, P., and Pannell, J. (2022). Recurrent allopolyploidization, Y-chromosome introgression and the evolution of sexual systems in the plant genus Mercurialis. Phil. Trans. R. Soc. B **377,** 20210224.

Harkess, A., Huang, K., Hulst, R.V.d., Tissen, B., Caplan, J.L., Koppula, A., Batish, M., Meyers, B.C., and Leebens-Mack, J.H. (2020). Sex determination by two Y-linked genes in garden asparagus. Plant Cell **32,** 1790–1796.

Havill, J.S., Richardson, B.J., Rohwer, C.L., Gent, D.H., Henning, J.A., and Muehlbauer, G.J. (2023). Identification of quantitative trait loci associated with R1-mediated resistance to powdery mildew and sex determination in hop (Humulus lupulus L.). Theor. Appl. Genet. **136**.

Hough, J., Hollister, J.D., Wang, W., Barrett, S.C.H., and Otto, S.P. (2014). Genetic degeneration of old and young Y chromosomes in the flowering plant *Rumex hastatulus* . Proc. Natl. Acad. Sci. U. S. A. **111,** 7713–7718.

Jesionek, W., Bodláková, M., Kubát, Z., Čegan, R., Vyskot, B., Vrána, J., Šafář, J., Puterova, J., and Hobza, R. (2021). Fundamentally different repetitive element composition of sex chromosomes in *Rumex acetosa* . Ann. Bot. **127,** 33–47.

Jia, H.M., Jia, H.J., Cai, Q.L., Wang, Y., Zhao, H.B., Yang, W.F., Wang, G.Y., Li, Y.H., Zhan, D.L., Shen, Y.T., Niu, Q.F., Chang, L., Qiu, J., Zhao, L., Xie, H.B., Fu, W.Y., Jin, J., Li, X.W., Jiao, Y., Zhou, C.C., Tu, T., Chai, C.Y., Gao, J.L., Fan, L.J., van de Weg, E., Wang, J.Y., and Gao, Z.S. (2019). The red bayberry genome and genetic basis of sex determination. Plant Biotechnology Journal **17,** 397-409.

Käfer, J., Bewick, A., Andres-Robin, A., Lapetoule, G., Harkess, A., Caïus, J., Fogliani, B., Gâteblé, G., Ralph, P., dePamphilis, C.W., Picard, F., Scutt, C., Marais, G.A.B., and Leebens-Mack, J. (2021). A derived ZW chromosome system in Amborella trichopoda, representing the sister lineage to all other extant flowering plants. New Phytol.

Li, H., Jia, K.-H., Zhang, R.-G., Wang, Y., Shi, T.-L., Z-C Li, Zeng, S.-W., X-J Cai, Wagner, N., Hörandl, E., Muyle, A., Yang, K., Charlesworth, D., and Mao, J.-F. (2021). Chromosome-scale assembly of the genome of Salix dunnii reveals a male-heterogametic sex determination system on chromosome 7. Molecular Ecology Resources.

Liu, Z., Moore, P.H., Ma, H., Ackerman, C.M., Ragiba, M., Pearl, H.M., Kim, M.S., Charlton, J.W., Yu, Q., Stiles, J.I., Zee, F.T., Paterson, A.H., and Ming, R. (2004). A primitive Y chromosome in Papaya marks the beginning of sex chromosome evolution. Nature **427,** 348-352.

Massonnet, M., Cochetel, N., Minio, A., A, M.V., Muyle, A., Lin, J., Garcia, J.F., Zhou, Y., Delledonne, M., Riaz, S., Figueroa-Balderas, R., Gaut, B.S., and Cantu, D. (2020). The genetic basis of sex determination in grapevines (Vitis spp.). Nature Communications **11,** :2902.

Masuda, K., Ikeda, Y., Matsuura, T., Kawakatsu, T., Tao, R., Kubo, Y., Ushijima, K., Henry, I.M., and Akagi, T. (2022). Reinvention of hermaphroditism via activation of a RADIALIS-like gene in hexaploid persimmon. Nature Plants **8,** 217-+.

Müller, N., Kersten, B., Montalvão, A., Mähler, N., Bernhardsson, C., Bräutigam, K., Lorenzo, Z., Hoenicka, H., Kumar, V., Mader, M., Pakull, B., Robinson, K.M., Sabatti, M., Vettori, C., Ingvarsson, P., Cronk, Q., Street, N., and Fladung, M. (2020). A single gene underlies the dynamic evolution of poplar sex determination. Nature Plants **6,** 630–637.

Ngwe, F.N., and Siljak-Yakovlev, S. (2023). Sex Determination in Dioscorea dumetorum: Evidence of Heteromorphic Sex Chromosomes and Sex-Linked NORs. Plants-Basel **12**.

Palmer, W., Jacygrad, E., Sagayaradj, S., Cavanaugh, K., Han, R., Bertier, L., Beede, B., Kafkas, S., Golino, D., Preece, J., and Michelmore, R. (2022). Genome assembly and association tests identify interacting loci associated with vigor, precocity, and sex in interspecific pistachio rootstocks. G3 Genes|Genomes|Genetics **13**.

Picq, S., Santoni, S., Lacombe, T., Latreille, M., Weber, A., Ardisson, M., Ivorra, S., Maghradze, D., Garcia-Arroyo, R., This, P., Terral, J.-F., and Bacilieri, R. (2014). A small XY chromosomal region explains sex determination in wild dioecious *V. vinifera* and the reversal to hermaphroditism in domesticated grapevines. BMC Plant Biology **14,** 229.

Prentout, D., Stajner, N., Cerenak, A., Tricou, T., Brochier-Armanet, C., Jakse, J., Käfer, J., and Marais, G.A.B. (2021). Plant genera Cannabis and Humulus share the same pair of well-differentiated sex chromosomes. New Phytol. **231,** 1599-1611.

Sanderson, B., Feng, G., Hu, N., Carlson, C., ..., Ma, T., Liu, J., DiFazio, S., and Olson, M. (2021). Sex determination through X-Y heterogamety in *Salix nigra* . Heredity **126,** v.

She, H., Xu, Z., Zhang, H., Wu, J., Wang, X., Liu, Z., and Qian, W. (2022). Remarkable divergence of the sex-linked region between two wild spinach progenitors, *Spinacia turkestanica* and *Spinacia tetrandra* . Biology (Basel) **11**.

Sola-Campoy, P.J., Robles, F., Schwarzacher, T., Ruiz Rejón, C., de la Herrán, R., and Navajas-Pérez, R. (2015). The molecular cytogenetic characterization of pistachio (*Pistacia ver* a l.) suggests the arrest of recombination in the largest heteropycnotic pai. PLOS ONE **10,** e0143861.

Sousa, A., Fuchs, J., and Renner, S. (2017). Cytogenetic comparison of heteromorphic and homomorphic sex chromosomes in Coccinia (Cucurbitaceae) points to sex chromosome turnover. Chromosome Res. **25,** 191-200.

Tamiru, M., Natsume, S., Takagi, H., White, B., Yaegashi, H., Shimizu, M., Yoshida, K., Uemura, A., Oikawa, K., Abe, A., Urasaki, N., Matsumura, H., Babil, P., Yamanaka, S., Matsumoto, R., Muranaka, S., Girma, G., Lopez-Montes, A., Gedil, M., Bhattacharjee, R., Abberton, M., Kumar, P.L., Rabbi, I., Tsujimura, M., Terachi, T., Haerty, W., Corpas, M., Kamoun, S., Kahl, G., Takagi, H., Asiedu, R., and Terauchi, R. (2017). Genome sequencing of the staple food crop white Guinea yam enables the development of a molecular marker for sex determination. BMC Biology **15,** 86.

Tennessen, J.A., Govindarajulu, R., Liston, A., and Ashman, T.-L. (2016). Homomorphic ZW chromosomes in a wild strawberry show distinctive recombination heterogeneity but a small sex-determining region. New Phytol. **211**.

Torres, M., Mathew, L.S., Ahmed, I., Al-Azwan, I.K., Krueger, R., Rivera-Nuñez, D., Mohamoud, Y.A., Clark, A.G., Suhre, K., and Malek, J.A. (2018). Genus-wide sequencing supports a two-locus model for sex-determination in Phoenix. Nature Communications **9,** 3969.

Wang, D., Li, Y., Li, M., Yang, W., Ma, X., Zhang, L., Wang, Y., Feng, Y., Zhang, Y., Zhou, R., Sanderson, B.J., Keefover-Ring, K., Yin, T., Smart, L.B., DiFazio, S.P., Liu, J., Olson, M., and Ma, T. (2022). Repeated turnovers keep sex chromosomes young in willows. Genome Biology **23,** 200.

Wang, J., Na, J., Yu, Q., Gschwend, A.R., Han, J., Zeng, F., Aryal, R., VanBuren, R., Murray, J.E., Zhang, W., Navajas-Pérez, R., Feltus, F., Lemke, C., Tong, E.J., Chen, C., Wai, C., Singh, R., Wang, M., Min, X., Alam, M., Charlesworth, D., Moore, P.H., Jiang, J., Paterson, A.H., and Ming, R. (2012). Sequencing papaya X and Yh chromosomes reveals molecular basis of incipient sex chromosome evolution. Proc. Natl. Acad. Sci. U. S. A. **109,** 13710-13715.

Wu, M., Haak, D.C., Anderson, G.J., Hahn, M.W., Moyle, L.C., and Guerrero, R.F. (2021). Inferring the genetic basis of sex determination from the genome of a dioecious nightshade. Mol. Bio. Evol. **38,** 2946-2957.

Xue, L., Wu, H., Chen, Y., Li, X., Hou, J., Lu, J., S Wei, Dai, X., Olson, M.S., Liu, J., Wan, M., Charlesworth, D., and Yin, T. (2020). Evidences for a role of two Y-specific genes in sex determination in *Populus deltoides* . Nature Communications **11,** 5893.

Yue, J., Krasovec, M., Kazama, Y., Zhang, X., Xie, W., Zhang, S., Xu, X., Kan, B., Ming, R., and Filatov, D.A. (2023). The origin and evolution of sex chromosomes, revealed by sequencing of the *Silene latifolia* female genome. Curr. Biol. **33,** 2504-2514.e2503.

Zhou, R., Macaya-Sanz, D., Rodgers-Melnick, E., Carlson, C., Gouker, F., Evans, L., Schmutz, J., Jenkins, J.W., Yan, J., Tuskan, G.A., Smart, L.B., and DiFazio, S.P. (2019). Characterization of a large sex determination region in *Salix purpurea* L. (Salicaceae). Mol. Genet. Genomics **293,** 1437-1452.

Zhou, R., Macaya-Sanz, D., Carlson, C., Schmutz, J., Jenkins, J.W., Kudrna, D., Sharma, A., Sandor, L., Shu, S., Barry, K., Tuskan, G.A., Ma, T., Liu, J., Olson, M., Smart, L.B., and DiFazio, S.P. (2020). A willow sex chromosome reveals convergent evolution of complex palindromic repeats. Genome Biology **21,** 38.
